# Seroprevalence of *Toxoplasma gondii* infection in Norwegian dairy goats

**DOI:** 10.1186/1751-0147-54-75

**Published:** 2012-12-21

**Authors:** Marit Stormoen, Jorun Tharaldsen, Petter Hopp

**Affiliations:** 1 Norwegian School of Veterinary Science, P.O. Box 8146 Dep, NO-0033, Oslo, Norway; 2 Norwegian Veterinary Institute, P.O. Box 750 Sentrum, NO-0106, Oslo, Norway

**Keywords:** *Toxoplasma gondii*, Goats, Norway, Prevalence, Serology

## Abstract

**Background:**

*Toxoplasma gondii* is a major problem for the sheep industry as it may cause reproduction problems. The importance of *T. gondii* in Norwegian goat herds is uncertain, but outbreaks of toxoplasmosis in dairy goat farms have been recorded. The aim of this study was to describe the prevalence of *T. gondii* infection in Norwegian dairy goats by using serology.

**Findings:**

Goat serum originally collected as part of two nationwide surveillance and control programmes between 2002 and 2008 were examined for *T. gondii* antibodies by using direct agglutination test. In total, 55 of 73 herds (75%) had one or more serologically positive animals, while 377 of 2188 (17%) of the individual samples tested positive for *T. gondii* antibodies.

**Conclusions:**

This is the first prevalence study of *T. gondii* infection in Norwegian goats. The results show that Norwegian goat herds are commonly exposed to *T. gondii*. Nevertheless, the majority of goat herds have a low prevalence of antibody positive animals, which make them vulnerable to infections with *T. gondii* during the gestation period.

## Findings

*Toxoplasma gondii* is a coccidian parasite which has felids as the definitive host and all warm-blooded animals, including humans, are potential intermediate hosts [1]. *T. gondii* is a potential problem for the goat and sheep husbandry as it may cause abortions, stillbirths and neonatal death. The importance of *T. gondii* infection in Norwegian goat husbandry is uncertain. In a study of foetal loss, toxoplasmosis was indicated as the cause of 7 of 160 abortions (4.3%) [2], but during an outbreak of toxoplasmosis in a dairy goat farm, around 70% of the animals experienced abortions, stillbirths or did not conceive [3].

In 2007, the Norwegian goat population was 71,500 goats, of which 41,000 were dairy goats distributed among 490 herds (the Register of Production Subsidies as of 31^st^ July 2007, Norwegian Agricultural Authority, Oslo). The aim of this study was to describe the seroprevalence of *T. gondii* infection in Norwegian dairy goats.

The blood samples used had previously been collected as part of two different surveillance and control programmes. Source A consisted of material collected within the national surveillance programme for *Brucella melitensis*[4]. Samples collected from dairy goat herds between September and December in 2008, in total 21 herds, were available for this study. From each of these herds, serum samples from 30 animals at least one year old were examined for *T. gondii*, except from one herd where only 28 animals had been bled.

Source B comprised material from 569 different goat herds that had been collected from 2002 to 2008 within the disease eradication programme Healthier goats [5]. The herds were stratified into three regions, East (Østfold, Akershus, Oslo, Hedmark, Oppland, Buskerud, Vestfold, Telemark, Aust-Agder, Vest-Agder), West (Rogaland, Hordaland, Sogn og Fjordane, Møre og Romsdal) and, North (Sør-Trøndelag, Nord-Trøndelag, Nordland, Troms, Finnmark), and the proportion of goat herds in each region compared to the whole population determined the number of herds selected. A random selection from each region was performed, with the modification that at least two herds should be selected from each county with dairy goats. A total of 52 herds with samples from at least 30 individuals one year old or older were selected from source B. From each of these herds, samples from 30 animals were examined for *T. gondii*. If more than 30 samples were available, the most recent samples from the oldest animals were chosen.

In total, 2188 serum samples from 73 goat herds were included in the study.

The serum samples, which had been stored at −20°C, were thawed and thereafter examined for *T. gondii* specific IgG antibodies using a commercial direct agglutination test (Toxo-Screen DA, bioMerieux, France) in the dilution 1:40. Sera with a positive reaction were regarded as seropositive. The herd was classified as positive for *T. gondii* when at least one goat within the herd was seropositive.

The herd-level and individual-level prevalences and the corresponding confidence intervals were estimated by using the function svymean (“survey: analysis of complex survey samples”, R package version 3.28-2, 2012, Lumley T. [6]) in R (R 2.15.1 for Windows, 2012, the R Foundation for Statistical Computing [7]). The herd was the primary sampling unit, individual level prevalences were estimated taking clustering into consideration and stratification by region were included when analysing data from source B.

Differences in serological response between regions or sample sources were investigated by univariable and multivariable logistic regression using glmmPQL (R-package MASS version 7.3-20 [8]) in R. The individual was the statistical unit, the serological response was the outcome, region and/or sample source were the explanatory variables, and herd was included as a random effect. A significance level of 0.05 was chosen.

**Figure 1:**
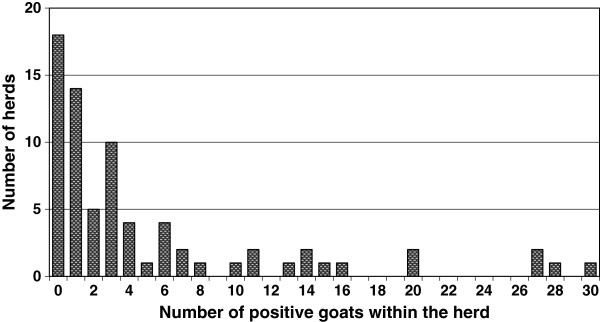
**The number of goats within a herd that were serologically positive for*Toxoplasma gondii *in a study in Norway in 2009.** The study comprised 73 goat herds and from each herd 30 animals were examined, except one herd where only samples from 28 individuals were available.

There was at least one serologically positive animal in 55 (75%) herds. The prevalence of herds positive for *T. gondii* antibodies was 81% [64%-98%] (95% confidence interval) and 73% [61%-84%] for source A and source B, respectively. Within the positive herds the number of positive animals varied from 1 to 30 (3.3% to 100%) (Figure 1) and in 29 (53%) of these herds, one to three serologically positive animals were found.

A total of 377 (17%) individuals were serologically positive. The individual prevalence was 25% [12%-37%] and 14% [9.0%-19%] for source A and source B, respectively. No significant differences in the prevalence estimates were found between regions and/or sample sources.

The results of this study show that exposure of Norwegian goat herds to *T. gondii* is common. In Norwegian sheep, a prevalence of 44% seropositive flocks has previously been found [9]. However, in the sheep study another serological test was used and two positive animals were required before classifying the flock as positive. Hence, these prevalences cannot be directly compared and does not necessarily imply that Norwegian goat herds are more commonly exposed to *T. gondii* than Norwegian sheep flocks. In Europe, the reported individual prevalences of *T. gondii* antibodies in goats vary considerably from 12% to 69% [reviewed by 10]. Although these prevalences should be compared with caution due differences in study designs and serological tests used, the individual prevalence found in Norwegian goats is within the range of the prevalences reported in these European studies.

The finding that in more than 50% of the herds less than 10% of the tested animals were seropositive, indicates that a high proportion of the herds is at risk of reproductive problems caused by primary *T. gondii* infection during the pregnancy period. Goats in Norway are usually housed around the time of mating in autumn and thereafter confined indoors during winter. During the indoor period, when the gestation usually takes place, the animals are more likely to come into contact with cat faeces and may be exposed to feed contaminated by oocysts. Although rarely reported, abortion storms in Norwegian goats due to *T. gondii* infections have occasionally been observed [3], underlining the importance of precautionary measures to avoid *T. gondii* infection during the vulnerable gestation period.

*T. gondii* is a zoonotic infection and the parasite is usually transmitted to humans through ingestion of raw or undercooked meat from infected animals and through contaminated food and water [10,11]. In Norway, neither goat meat nor goat milk are commonly consumed. Nevertheless, one should be aware that undercooked goat meat and unpasteurized goat milk are potential sources of *T. gondii* infection [10,12].

In this study a commercial direct agglutination test developed for humans was used and the test sensitivity and specificity have not been reported for goat serum. However, the direct agglutination test and the modified agglutination test have previously been found to have comparable sensitivity on goat sera [13]. We used the same cut off as suggested for humans in the manufacturer’s instruction (1:40). The manufacturer recommends testing the sera in 1:4000 dilution to avoid false negative due to the prozone phenomenon in low dilutions of strongly positive sera. To reduce costs, this was not performed thereby potentially underestimating the seroprevalences.

This is the first prevalence study of antibodies to *T. gondii* in Norwegian goats, and we are not aware of any previous published studies on prevalence in Scandinavian goats. The results show that Norwegian goat herds are commonly exposed to *T. gondii*. Nevertheless, the majority of goat herds have a low prevalence of antibody positive animals, which make them vulnerable to infections with *T. gondii* during the gestation period.

## Competing interests

The authors declare none competing interests.

## Author’s contributions

MS designed the study, performed the analysis of the blood samples, the statistical analysis and drafted the first version of the manuscript. JT initiated the study and supervised the analysis of the blood samples. PH supervised in the design of the study and the epidemiological analysis. All authors have read and accepted the final manuscript.
